# From Malthusian Disequilibrium to the Post-Malthusian Era: The Evolution of the Preventive and Positive Checks in Germany, 1730–1870

**DOI:** 10.1007/s13524-020-00872-w

**Published:** 2020-05-04

**Authors:** Ulrich Pfister, Georg Fertig

**Affiliations:** 1 University of Münster, Institute of Economic and Social History, Domplatz 20-22, 48143 Münster, Germany; 2 University of Halle-Wittenberg, Institute of History, Steintorcampus, 06099 Halle (Saale), Germany

**Keywords:** Malthusian population dynamics, Population and development, Unified growth theory, Post-Malthusian regime

## Abstract

This study draws on a new data set of vital rates and real wages to explore short-term and long-term behavior of the preventive and positive checks in a major economy of premodern mainland Europe. Four results stand out. First, the preventive check was fairly stable throughout the period 1730–1870; its magnitude of 0.2 to 0.35 was comparable with that of England, northern and central Italy, and Sweden. Second, the eighteenth century was characterized by Malthusian disequilibrium in that there was no long-term relationship between the crude death rate and the real wage, whereas the crude death rate’s instantaneous response to income changes was a substantial –0.4. Third, the short-term positive check may have weakened over the eighteenth century and largely disappeared in the 1810s. The diversification of food risk resulting from the spread of potato cultivation, market integration, and the development of the nonagricultural sectors are potential explanations of the demise and disappearance of the positive check. Fourth, between the 1810s and the 1860s, vital rates and the real wage were stationary, which is consistent with a post-Malthusian regime in which technological progress depended on population size. The 1810s marked the time when Germany transited from a Malthusian regime in disequilibrium to the post-Malthusian era.

**Electronic supplementary material:**

The online version of this article (10.1007/s13524-020-00872-w) contains supplementary material, which is available to authorized users.

## Introduction

This study combines several novel data sets to analyze the interaction between vital rates and the real wage in pre-industrial and early industrial Germany. The focus is on the evolution of the Malthusian checks measured as the short-term and long-term elasticity of the crude death rate and the crude birth rate, respectively, on the real wage. Such an investigation speaks to two strands of recent demographic research.

First, our analysis is part of a second generation of empirical studies into Malthusian relationships using historical demographic data. A first generation of studies, published mostly in the 1980s, studied mainly the short-term bivariate relationships between vital events or vital rates and grain prices as a proxy for income (e.g., Galloway 1988; Lee 1981; Weir 1984). More recent work, by contrast, has made use of new or improved data sets of vital rates and real wages and has employed multivariate time series methods—most notably, vector autoregression (VAR) and structural time series analysis—to study Malthusian relationships from a systemic perspective (e.g., Edvinsson 2017; Fernihough 2013; Lee and Anderson 2002; Møller and Sharp 2014; Nicolini 2007). This change in methodology has also led to a greater attention for structural, long-term interaction between demographic and economic variables as opposed to short-term relationships. In this study, we join this second-generation research into the empirics of Malthusian relations by introducing a new data set of vital rates for a major European economy reaching back to 1730. Whereas English vital rates and real wages display unit roots (Bailey and Chambers 1993:350–351), we find that the death rate was either stationary or trend-stationary, depending on the period considered. This finding implies that the identification of Malthusian dynamics and the transition to a post-Malthusian regime must consider both short-term and long-term structural relationships. Given the time series properties of our data, we opt for a flexible combination of autoregressive distributed lag regression and error correction mechanism (ECM) analysis. Beyond the specific case of pre-industrial and early industrial Germany, our approach may provide a suitable strategy to analyze other empirical situations characterized by a simultaneous presence of stationary and integrated series, such as Italy before circa 1880 (cf. Fernihough 2013:323).

Second, our work contributes to the stock of stylized facts that underlie models of the long-term evolution of the relationship between demographic variables and economic development on the macro level, particularly in the form of unified growth theory (e.g., Galor 2011; Galor and Weil 2000; Kremer 1993). Specifically, we establish that during 1815–1870, Germany corresponded well to major aspects of a post-Malthusian regime. A positive relationship between the level of technology and population fully compensated for the negative impact of population size on material welfare. Population growth was twice as fast as in the eighteenth century, consistent with the idea that in a situation characterized by low demand for human capital, the primary effect of technological progress is to ease household budget constraints, which leads to raising more children. The transition to the post-Malthusian era took place quite suddenly in the late 1800s and 1810s and was predated by Malthusian disequilibrium in two respects. First, during 1730–1799, the real wage followed a steeply falling trend without Malthusian adjustment setting in. An important reason for this finding is, second, the absence of a long-term relationship between the real wage and the death rate. Because the birth rate adjusted only partially for income fluctuations, population could expand despite a fall of the real wage, which resulted in a high vulnerability of the death rate with respect to short-term supply shocks. The disequilibrium found in eighteenth century Germany may have been typical for wider parts of mainland Europe during the early modern period. In eight countries (excluding England, the United Provinces, and Belgium), population almost doubled between 1500 and 1800 (plus 89%; Allen 2000:8–9), whereas the real wage fell by about one-half (Allen 2001:428–430), suggesting that effective adjustment to a Malthusian equilibrium wage was weak or absent.

The findings for eighteenth century Germany do not tally well with an endogenous model of the transition from the Malthusian to the post-Malthusian era. Galor and Weil (2000; see also Galor 2011:149–168; Kremer 1993) assumed a positive relationship both between population size and the rate of technological progress as well as between investment in human capital and the rate of expected technological progress. Moreover, there is a threshold level of the rate of technological progress below which investment in human capital is nil. The Malthusian era is then characterized by slow technological progress and slow population growth. Over time, the rate of technological progress accelerates and crosses the threshold beyond which investment in human capital is feasible. The result is a post-Malthusian phase in which technology rises faster than population growth and parents reduce the number of children because they shift their demand from quantity to quality. The steep fall of the real wage in eighteenth century Germany renders it unlikely that this economy experienced a positive interaction between population size and the rate of technological change in the century before the transition to the post-Malthusian era. Nevertheless, the population growth accompanying disequilibrium may still have laid the basis for a continuous stream of size-dependent technological advances after 1800. For this reason, the interpretation of our results will consider both endogenous and exogenous factors in Germany’s transition to the post-Malthusian era.

## Issues in the Analysis of Malthusian Systems

Most empirical analyses of Malthusian population dynamics capture Malthusian theory with the following set of equations (e.g., Fernihough 2013:313–314; Lee and Anderson 2002:205–207; Møller and Sharp 2014:109–111):

1 $$ {w}_t={\upalpha}_t-\upbeta {p}_t $$

2 $$ {b}_t={c}_1+\upgamma {w}_t+{u}_{bt} $$

3 $$ {d}_t={c}_2-\updelta {w}_t+{u}_{dt} $$

4 $$ {p}_t={p}_{t-1}+{b}_{t-1}-{d}_{t-1}, $$

where *w*_*t*_ is the natural logarithm of the real wage, *b*_*t*_ is the crude birth rate, *d*_*t*_ is the crude death rate, α_*t*_ is the level of technology, and *p*_*t*_ is the natural logarithm of population size. β is the elasticity of the real wage on population size; given diminishing returns of output with respect to population, its value is less than 1. In a standard Cobb-Douglas framework with two factors of production, of which one is fixed (e.g., land), (1 − β) is equal to the weight of labor in the production function and to the share of labor in total factor income, respectively. γ is the coefficient of the preventive check, and δ is the coefficient of the positive check. Finally, *u*_*bt*_ and *u*_*dt*_ model unsystematic exogenous shocks.

Equation (1) can be read as a labor demand schedule whose level depends positively on technology and where population proxies the labor input. Equations (2) and (3) describe demographic adjustment to shocks to the real wage. Specifically, the preventive check in Eq. (2) consists in a positive reaction of the birth rate to an increase of the real wage, whereas the positive check in Eq. (3) means that the death rate declines in reaction to an increase in the real wage. Equation (4) feeds Malthusian adjustment back into population by stating that in a closed economy with zero migration, the rate of population growth equals the rate of natural increase.

Taken together, Eqs. (1)–(4) define a Malthusian equilibrium conditional on the level of technology ($\overline{\upalpha}$). Specifically, with given technology the equilibrium values (denoted by asterisks) of the real wage, the vital rates and of population are

5 $$ {w}^{\ast }=\left({c}_2-{c}_1\right)/\left(\upgamma +\updelta \right) $$

6 $$ {b}^{\ast }={d}^{\ast }=\left({c}_2\upgamma +{c}_1\updelta \right)/\left(\upgamma +\updelta \right) $$

7 $$ {p}^{\ast }=\raisebox{1ex}{$1$}\!\left/ \!\raisebox{-1ex}{$\upbeta $}\right.\left(\upalpha --\kern0.30em {w}^{\ast}\right). $$

Take a shock in *u*_*dt*_ in the form of an epidemic, such as plague, which drastically increases the death rate for a moment (Eq. (3)). Consequently, population declines (Eq. (4)) and the real wage increases (Eq. (1)). Through the Malthusian checks of Eqs. (2) and (3), the real wage increase translates into a higher birth rate and a lower death rate. As a result, population increases (Eq. (4)) and over successive periods drives population and the real wage back to their respective equilibrium values. Because the level of technology impacts on p* but not on w*, long-run technological change at low levels leads to an increase in population but not in the real wage (Eqs. (5) and (7)). This is the situation prevailing around the year 1500. Regions with high levels of agricultural technology were characterized by high population density but not by higher welfare levels in comparison with marginal regions (Ashraf and Galor 2011; Galor 2011: chapter 3). Subsequent real wage divergence (Allen 2001) may thus reflect differences with respect to the moment when economies transited from Malthusian to a post-Malthusian regime. Thus, an investigation into this transition holds the potential to provide relevant insights into the forces that produced modern economic development.

Even under a Malthusian regime, technological progress is possible, of course. Denote technological growth with *g*_*t*_ = α_*t*_ − α_*t*  −  1_. As long as *g*_*t*_ remains sufficiently low, Malthusian adjustment fully compensates for technological progress. Consequently, the real wage and the vital rates are stationary around their equilibrium values given in Eqs. (5) and (6). In this case, Eqs. (1)–(4) can be analyzed with a VAR of the multivariate series (*b*_*t*_, *d*_*t*_, *w*_*t*_) (Eckstein et al. 1984; cf. Møller and Sharp 2014:113–115; Nicolini 2007).

Matters become more complicated when technological progress accelerates. It is reasonable to assume that the roles of institutional change and knowledge accumulation in technological progress contribute to a data-generating process in which the level of technology at a given year is dependent on the level of technology in the previous year plus some random deviation. In such a situation, the unobserved variable α_*t*_ has a unit root. If the influence of α_*t*_ on the real wage prevails over the one of population in Eq. (1) either because of its magnitude or because of a weakening of the Malthusian checks, then the time series properties of α_*t*_ translate to those of the real wage and the vital rates. Accordingly, these variables also have a unit root, and there are two cointegrating relationships in the system: namely, between the real wage and each of the vital rates. Because the preventive and the positive checks operate independently, there is no cointegration between the birth and the death rate (Møller and Sharp 2014:116–118; cf. also Bailey and Chambers 1993). In this situation, the estimation of structural time series models (Lee and Anderson 2002) and use of cointegrated VAR (Møller and Sharp 2014) are appropriate empirical strategies. Specifically, Møller and Sharp (2014:123–124), using the second method, showed that by the 1620s, the English economy was non-Malthusian in that the system of the vital rates was not stationary but included cointegrating relationships.

A post-Malthusian situation can also emerge in another way, however. Assume that the Malthusian checks are weak or nonexistent (γ ≈ δ ≈ 0) such that population grows at the average rate of *c*_1_ − *c*_2_. If technology is related to population size such that its equilibrium growth rate (*g*^∗^) is *g*^∗^ = β(*c*_1_ − *c*_2_), then technological progress compensates for the negative effect of population on the real wage. Consequently, stationary real wage and vital rates follow, and population is trend-stationary. Scale-dependent technology, which leads to an association between the level of technology and population size, is suggested by both Smithian and Boserupian views on economic development. Population growth increases market size, which encourages specialization along comparative advantage, thereby improving factor allocation and increasing the technical efficiency of the economy (Kelly 1997). In a Boserupian perspective, population growth facilitates the circulation of information and thereby contributes to the spread of labor-intensive technical innovations in agriculture (Boserup 1965). Our results suggest that during the half-century preceding national unification in 1871, Germany followed the pattern of a post-Malthusian regime with scale-dependent technology. Whereas a positive relationship between technology and population appears thus as a plausible ingredient of the transition out of a Malthusian regime, there is no theoretical justification for the condition *g*^∗^ = β(*c*_1_ − *c*_2_) to hold. If equilibrium occurs, it is fortuitous and potentially unstable.

Finally, it is empirically possible that some of the variables of interest have a unit root but others are stationary. Italy is an example (Fernihough 2013:323), and later in the article we show that the same holds for Germany before circa 1800. These findings contrast with the English case, where all vital series have a unit root (Bailey and Chambers 1993:350–351). When some variables have a unit root but others are stationary, the real wage and the vital rates do not constitute a stable Malthusian system in terms of Eqs. (1)–(7). To the extent that technological progress is slow such that population has a negative impact on material welfare, we can still characterize such a situation as Malthusian (Eq. (1); cf., e.g., Clark 2007:20), but it is one characterized by disequilibrium because it lacks the mechanisms to reach the equilibrium state described by Eqs. (5)–(7).

The simultaneous presence of stationary and integrated variables also renders it impossible to investigate Eqs. (1)–(3) with a single method. Hence, our approach is to estimate the two Malthusian checks separately using standard methodology to estimate demand elasticities for individual goods from time series (Cuddington and Dagher 2015). Our baseline model is an equation with an ECM between two variables having a unit root

8 $$ \Delta  {r}_t=c+\uplambda \left({r}_{t-1}-{\upvarepsilon}_w{w}_{t-1}\right)+{v}_{w0}\Delta  {w}_t+\sum \limits_{r=1}^{R-1}{v}_{w,r}\Delta {w}_{t-r}+\sum \limits_{l=1}^{L-1}{v}_{r,l}\Delta  {r}_{t-l}+{u}_t, $$

where *r*_*t*_ is a vital rate (either *b*_*t*_ or *d*_*t*_,), *w*_*t*_ is the natural logarithm of the real wage as earlier, *R* and *L* refer to lag orders, and *u*_*t*_ is an i.i.d. error term; the remainder are parameters. Following the general logic of cointegration and ECM analysis, Eq. (8) produces estimates for the long-term and the short-term elasticities as well as the speed with which a deviation from the long-term relationship is corrected (cf. Brooks 2002:390–391; Greene 2012:959, 963–964; Lütkepohl 2007:246–247). The coefficient of the contemporaneous effect *v*_*w0*_ indicates the central component of the short-term elasticity of a vital rate on the real wage (i.e., γ or δ in Eqs. (2) and (3)). Later in the article, we refer to *v*_*w0*_ as the instantaneous elasticity of a vital rate on the real wage. In addition, we employ the sum of *v*_*w*0_ and all *v*_*w*,*r* + 1_ to characterize the cumulative short-term elasticity of a vital rate on the real wage. In substantive terms, the magnitude of the short-term elasticity of vital events on the real wage is a measure of the vulnerability or resilience of a population with respect to shocks regarding the availability of economic resources and thus constitutes an aspect of the standard of living (cf. Bengtsson et al. 2004). In a Malthusian perspective, a high short-term elasticity particularly of the death rate with respect to the real wage indicates a great importance of short-term shocks, particularly famines, for the adjustment of population to the means of subsistence (Malthus 1798/1998:43–44).

By contrast, the long-term elasticity follows from the cointegrating coefficient ε_*w*_ in the error correction term. It refers to a structural relationship between vital rates and the real wage that holds beyond short-term shocks and across several cohorts. The coefficient of the error correction term, λ, indicates the proportion of a deviation from long-term equilibrium that is corrected in one period—in this case, corrected within a year. It indicates the speed at which a vital rate adjusts to a shock in the real wage and thus provides a measure of how fast Malthusian adjustment takes place.

The ECM of Eq. (8) has an equivalent formulation as an autoregressive distributed lag regression (ADL) (Cuddington and Dagher 2015:194–195, 203–206; for a multivariate situation, see Lütkepohl 2007:246–247):

9 $$ {r}_t={\upgamma}_0+{v}_{w0}{w}_t+\sum \limits_{r=1}^{R-1}{v}_{w,r}{w}_{t-r}+\sum \limits_{l=1}^{L-1}{v}_{r,l}{r}_{t-l}+{u}_t. $$

When both the vital rate and the real wage are stationary, ordinary least squares (OLS) regression of Eq. (9) yields consistent estimates of elasticity coefficients. First-generation work on the population dynamics of eighteenth and nineteenth century mainland Europe relied essentially on distributed lag (DL) or ADL regression, which thus turns out as appropriate empirical strategy when the underlying variables are stationary (see notably Galloway 1988; Lee 1981; Weir 1984). More generally, when a vital rate is stationary, we can obtain only estimates for its short-term elasticity on the real wage, which gives a sense of the vulnerability of a population with respect to shocks regarding the availability of economic resources.

The standard ECM and ADL regressions in Eqs. (8) and (9) consider only the bivariate relationship between a vital rate and the real wage. However, perhaps the other vital rate interferes with this relationship so that the elasticity estimate based on the bivariate relationship may suffer from omitted variable bias. To address this issue, we carry out exploratory reduced-form VAR analyses to explore whether Eqs. (8) and (9) should be augmented by effects of the other vital rate (discussed later in the section, Estimation of the Malthusian Checks). In sum, we flexibly combine ECM, ADL regression, and stationary VAR analysis to arrive at accurate estimates of the magnitudes of the preventive and positive checks (γ and δ in Eqs. (2) and (3)) in order to address a situation in which some variables are stationary while others have a unit root.

## Historical Overview and Data Sources

In this investigation, we bring together several new data series (see Table B1 in the online appendix). We start with a brief description of the data sources and the methods employed in producing the time series underlying our analysis. In a second step, we employ visual inspection of these data to characterize the evolution of the German economy and demography during the one and a half centuries prior to national unification in 1871.

*Vital rates* refer to an area comprising Germany with its present borders less South Schleswig and plus Eastern Belgium, Silesia and the parts of Pomerania and Brandenburg situated east of the Oder River. Historically, this area was part of both the early modern Empire and the nation state formed in 1871. From 1815, data rest on an exhaustive compilation of official sources documenting aggregate population and vital events for individual German states (Fertig et al. 2018). These new series systematically deal with underreporting during the earlier part of the nineteenth century and construct population figures between census years using vital events, whereas earlier research has relied on linear interpolation.

**Fig. 1:**
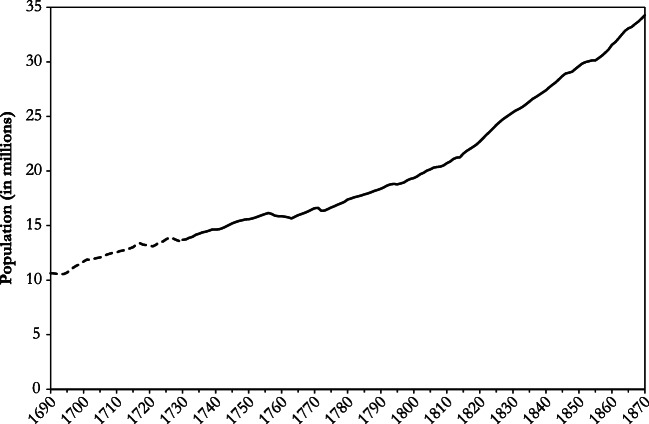
Population of Germany, 1690–1870 (in millions). *Sources*: Section B of the online appendix; original sources are Fertig et al. (2018:31–33, online appendix) and Pfister and Fertig (2010); cf. Historical Overview and Data Sources section in the text.

Aggregate figures for population and vital events for 1730–1815 rest on proto-statistical records of individual German territories and on published work relating to some 140 parishes (Pfister and Fertig 2010:9–10, 13–30). The bulk of the information is from Gehrmann’s (2000) study on northern Germany, which is augmented with material from other regions. Around 1790, coverage of vital events is about 30% of estimated national totals. As one moves back in time, this rate declines as sources compiled by state authorities diminish, and information from parish registers correspondingly gains in importance. Relatively frequent censuses render it possible to adjust population estimates for errors and net emigration back to about 1730. Tentative estimates exist for the period 1690–1730, but because of their poor internal consistency, we do not use them in this study except for the graph in Fig. 1. For years up to 1850, the *real wage* refers to day wages of unskilled males in urban construction from 18 towns (Pfister 2017). Using wages of urban construction workers follows current practice in the relevant literature because data availability is sufficient to construct annual series for only this type of workers and because payment in kind was negligible in the building trade. Nevertheless, given that employment in construction fluctuated considerably, urban construction interacted with labor markets for other occupations, so that urban building wages are representative for wider regions and a larger set of economic activities. Specifically, during circa 1730–1850, nominal wages of agricultural workers largely followed those of urban building laborers on the level of decades (Pfister 2017:710, Pfister 2019:233–234).

We deflate nominal silver wages of urban construction workers with the annual cost in grams of silver of a basket of 11 goods with fixed quantities following the method proposed by Allen (2001). The price information comes from 12 towns. In the 1760s, vegetable foods accounted for two-fifths of presumed household expenditures, with more than one-third relating to bread alone. Because grain prices were more volatile than prices of other products and because nominal wages were very sticky, short-term fluctuations in real wages are to a substantial part driven by fluctuations in grain prices (Pfister 2017:711–713, p. 1 of supporting information S2).

In 1850, we splice real wages of unskilled urban construction workers (which end in 1850) with a series of real wages of workers in industry and urban crafts that starts in the late 1840s (Pfister 2018). The consumer price index that serves to deflate nominal wages from 1850 is a Fisher index of the prices of broad categories of goods whose share in household expenditure is adjusted annually. The weight of vegetable foods in household expenditure declined linearly from 45% in 1849 to 29% in 1889 (Pfister 2018:579).

The Revolutionary and Napoleonic Wars (1792–1815) not only went together with major political, economic, and social upheaval, but also had a negative impact on the quality of our data series. Irregular data collection and the difficulty to aggregate data to territorial units with frequent boundary changes probably had an adverse effect on the quality of the population and vital events estimates for this period. Moreover, 1815 is an outlier: mortality decreased substantially in that year (see Fig. 3, lower panel). Finally, inflationary war finance reduced the reliability of the conversion factors underlying the computation of silver prices and wages. For all these reasons, when breaking down our data into subperiods, we omit the period 1800–1815, for which we suspect that the data series are of limited quality; we consider solely the eighteenth century (1730–1799) and the period from the Congress of Vienna to national unification (1816–1870).

Germany remained an overwhelmingly rural country until well into the nineteenth century. In 1871, about one-half of the workforce was active in agriculture, and the urbanization rate (the share of the population living in settlements with more than 5,000 inhabitants) rose above 10% only around 1800. Nevertheless, the development of regional export industries, which gained in momentum from the late seventeenth century, meant that a growing proportion of the rural population was engaged in nonagricultural activities. Around 1750, this share was at an order of magnitude of one-fifth; by 1850—a decade or so after the onset of the rapid development of modern industries—it had risen to about 38% (Pfister 2011:5). Thus, Germany was distinguished from the leading economies in northwestern Europe, France, and the northern half of Italy by an absence of premodern urban growth and limited structural change based on spatially dispersed handicraft industries. Only in the 1840s did rapid catch-up growth in industry set in (Tilly and Kopsidis forthcoming: chapter 8).

Despite slow structural change, Germany experienced considerable population growth during the period under study (Fig. 1). Already during the eighteenth century, population expanded at an annual rate of 0.4% (exponential trend in 1730–1799; all growth rates that follow are derived from exponential trend regression). After 1800, the pace of population growth accelerated to 0.8% in 1815–1870.

**Fig. 2:**
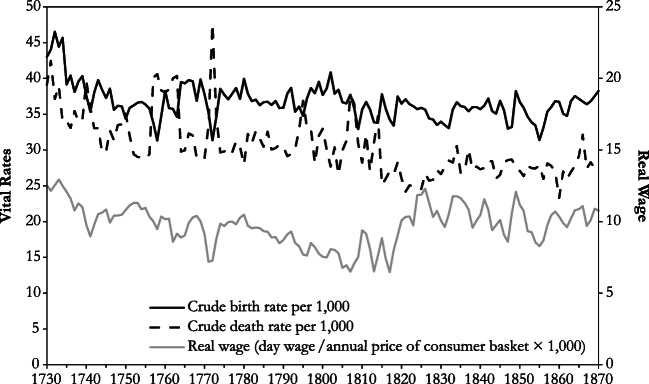
Vital rates (left axis) and real wage (right axis): Germany, 1730–1870. *Sources*: Vital rates: section B of the online appendix; original sources are Fertig et al. (2018:31–33) and Pfister and Fertig (2010). Real wage from Pfister (2017: supporting information S3, 2018: supplementary material A3); cf. Historical Overview and Data Sources section in the text.

Eighteenth century population growth is all the more remarkable given that the real wage fell at an annual rate of 0.5% in 1730–1799 (Fig. 2). This is a first indication that eighteenth century Germany was not in a Malthusian equilibrium. Whereas the negative link between population and material welfare was clearly in force, the real wage fell for a considerable time without effective adjustment taking place. Following the Revolutionary and Napoleonic Wars, the relationship between population and the real wage underwent drastic change. As Fig. 1 shows, the demographic effects of the wars were probably small; if anything, population growth increased.^1^ However, economic effects were considerable: the real wage reached its lowest level during the period under study in 1807, 1813, and 1817 (following the so-called Tambora crisis; Fig. 2). Postwar recovery and a series of bumper harvests in the 1820s, which benefitted workers’ living standards, quickly brought the real wage back to the level prevailing around the middle of the eighteenth century. Most remarkable of all is that it remained largely stable during the remainder of the period under study, despite an acceleration of population growth. Population could now expand at a rapid pace without a drastic fall in material conditions of life. Because this constitutes an important element of the post-Malthusian regime, the change of the relationship between the real wage and population growth gives a first indication that in Germany the Malthusian era ended in the 1810s.

Variations in fertility seem to have mattered little in the long-run evolution of population growth. After a decline from the extremely high values prevailing in the early 1730s, the level of the crude birth rate changed little during the remainder of the observation period. By contrast, the death rate fell between the 1730s and 1810s, which is surprising because on the background of a falling real wage, we would expect a decline of health conditions and hence a rise in mortality. Therefore, eighteenth century mortality decline must have originated from exogenous sources, and we explore some of these later in the article. The decline of the death rate was temporarily interrupted by episodes in which deaths exceeded births, which mostly reflect periods of European-wide famines (cf. Alfani et al. 2017:9–10; for a chronology based on German grain prices, see Albers et al. 2018:5–7). Such episodes, which went together with marked declines of the real wage, occurred in 1740, 1757/1758, 1761–1763 (both possibly in connection with the Seven Years’ War), 1772 (the worst mortality crisis on the national level during the period under study), 1795, possibly 1807/1808, and 1813/1814. Whereas poor regions with many land laborers, whose employment opportunities were negatively correlated with harvest outcomes, continued to experience subsistence crises with a negative rate of natural increase until 1855 (Bass 1991:42–46), such episodes did not occur on the national level anymore beginning with the so-called Tambora crisis of 1816/1817. This observation provides a second sign that a profound change of the demographic regime took place in the 1810s. Together, the eighteenth century mortality decline and the disappearance of episodes with excess mortality in the 1810s paved the way for the acceleration of population growth after 1815.

### Properties of the (*b*, *d, w*) Series

The remainder of this study explores the behavior of the multivariate time series composed of the birth rate *b*, the death rate *d*, and the real wage *w* over the period 1730–1870. The birth rate *b* and the death rate *d* are the vital rates as described in the previous section; the real wage *w* is the natural log of the real wage series shown in Fig. 2.

**Fig. 3:**
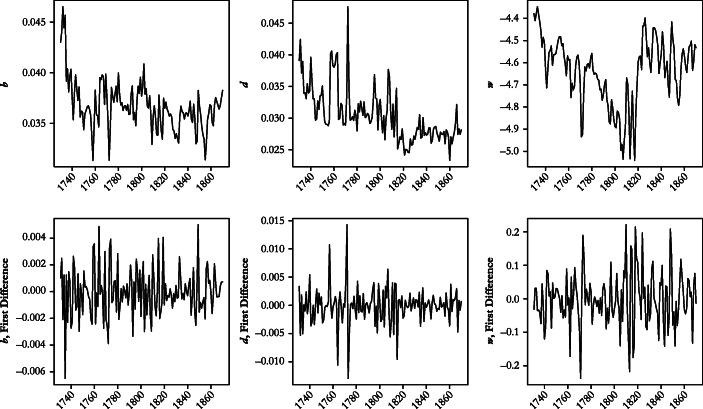
Data series: Crude birth rate (*b*), crude death rate (*d*), and natural logarithm of real wage (*w*), 1730–1870. The upper panel shows levels; the lower panel shows first differences. *Sources*: See Fig. 2 and the text.

Figure 3 shows the three series in levels (upper panel) and first differences (lower panel), respectively. The graphs for the first differences look quite regular, which suggests stationarity. The graphs for the levels, by contrast, look more irregular, which opens the possibility that one or more variables have a unit root and are thus integrated at the level *I*(1). To explore the time series properties of the multivariate (*b*, *d, w*) series in greater detail, section A of the online appendix reports the results of a battery of univariate unit root (augmented Dickey-Fuller (ADF)) and stationarity tests (Kwiatkowski-Phillips-Schmidt-Shin (KPSS)), and Table 1 establishes the rank of cointegration among the three variables. The unit root and stationarity tests produce a first substantive finding. For the period under study as a whole, the system (*b*, *d*, *w*) cannot have an equilibrium because the death rate is stationary or follows a declining trend, whereas tests for the other two series indicate that they are integrated at *I*(1). This implies that the forces driving the movement of the death rate are exogenous to the Malthusian system described by Eqs. (1)–(4).

**Table 1:** Johansen cointegration tests of crude birth rate (*b*), crude death rate (*d*), and log real wage (*w*)

		Critical Values		
Null Hypothesis	Trace Statistic	10%	5%	1%
Multivariate Series (*b*, *d*, *w*)				
1730–1870				
*r* = 0	87.02	39.06	42.44	48.45
*r* ≤ 1	34.86	22.76	25.32	30.45
*r* ≤ 2	7.10	10.49	12.25	16.26
1730–1799				
*r* = 0	58.50	39.06	42.44	48.45
*r* ≤ 1	24.07	22.76	25.32	30.45
*r* ≤ 2	8.50	10.49	12.25	16.26
1816–1870				
*r* = 0	59.29	7.52	9.24	12.97
*r* ≤ 1	31.29	17.85	19.96	24.60
*r* ≤ 2	9.55	7.52	9.24	12.97
Bivariate Series (*b*, *w*)				
1730–1870				
*r* = 0	38.81	22.76	25.32	30.45
*r* ≤ 1	6.50	10.49	12.25	16.26
1730–1799				
*r* = 0	28.35	22.76	25.32	30.45
*r* ≤ 1	6.13	10.49	12.25	16.26
Bivariate Series (*d*, *w*)				
1730–1870				
*r* = 0	54.09	22.76	25.32	30.45
*r* ≤ 1	14.59	10.49	12.25	16.26
1730–1799				
*r* = 0	38.66	22.76	25.32	30.45
*r* ≤ 1	12.16	10.49	12.25	16.26
Bivariate Series (*b*, *d*)				
1730–1870 (lag order = 3)				
*r* = 0	62.10	22.76	25.32	30.45
*r* ≤ 1	19.76	10.49	12.25	16.26
1730–1799				
*r* = 0	42.37	22.76	25.32	30.45
*r* ≤ 1	13.88	10.49	12.25	16.26

*Notes*: Tests for 1730–1870 and the subperiod 1730–1799 include a trend; tests for the subperiod 1816–1870 include a constant.

*Source*: Own calculation based on series shown in Fig. 3. Where not stated otherwise, lag order is 2.

That the death rate is trend-stationary in the whole period under study implies that there can be only one cointegrating relationship in the multivariate series (*b*, *d*, *w*): namely, between the birth rate and the real wage. Bivariate cointegration tests reported in the lower part of Table 1 indeed do not reject the null hypothesis of cointegrating rank equal to or less than 1 for the pair (*b*, *w*) in the whole period under study (1% level of statistical significance) and the subperiod 1730–1799 (5% level). The analogous tests for the relationships of the death rate with the birth rate and the real wage, by contrast, reject the null hypothesis of cointegration, usually at the 5% level of statistical significance.^2^ This is consistent with the fact that a stationary series cannot be part of a cointegrating relationship. The system (*b*, *d, w*) as a whole has a rank of 2 for the whole period under study and possibly also for the subperiod 1730–1799 (upper part of Table 1; for 1730, *r* ≤ 1 is rejected only at the 10% level of statistical significance). From the bivariate tests, it follows that one of the two cointegrating vectors captures the structural relationship between the birth rate and the death rate, whereas the other one is for the (stationary) death rate with zeros for all entries except for the death rate itself (Johansen 1995:37; Lütkepohl 2007:246, 250).

The finding that the system (*b*, *d, w*) is of cointegrating rank 2 rather than full rank in the whole period under study and for 1730–1799 replicates the findings reported elsewhere for preindustrial England (Møller and Sharp 2014:123–124) and nineteenth century Scandinavia (Klemp and Møller 2016:856). A major difference particularly between Germany and Britain, however, is that we find that the death rate was trend-stationary, whereas all vital rates in England were integrated at *I*(1) (Bailey and Chambers 1993:350–351; cf. Møller and Sharp 2014:121). Thus, in contrast to England, the system of vital rates and the real wage could not reach an equilibrium in Germany. As a structural relationship between the death rate and the real wage, the positive check (Eq. (3)) was not defined; forces exogenous to a Malthusian system drove the movement of mortality.

The pattern prevailing in 1816–1870 differs markedly from the one found for the whole period under study and for the subperiod 1730–1799. All three variables are stationary during the second subperiod (Table A1, online appendix). This implies that the system (*b*, *d, w*) is stationary or has full rank. Indeed, the test reported in Table 1 rejects the null hypothesis that its rank is equal to or less than 2 at the 5% level for the subperiod 1816–1870, implying that the cointegrating rank is 3, which corresponds to full rank. This finding should be related to the observation that population, while being integrated at *I*(1) or *I*(2) in 1730–1870, is trend-stationary in 1815–1870 (Fertig et al. 2018:19; Pfister et al. 2012:11–12). Stationarity of the vital rates and the real wage, and trend-stationarity of population, is consistent with a post-Malthusian situation in which the level of technology depends on population (as discussed earlier). This again contrasts with the British and Scandinavian experience, where the post-Malthusian era was characterized by nonstationarity of vital rates and the real wage (see Klemp and Møller 2016:856; Møller and Sharp 2014:123–124).

## Estimation of the Malthusian Checks

The results of the preceding section define the methods to analyze the relationships between the vital rates and the real wage. Because we find only one cointegrating relationship—the one between the birth rate and the real wage—we study the preventive check with a single-equation approach to an error correction model, as specified in Eq. (8). Trend-stationarity of the death rate, by contrast, renders possible the application of autoregressive distributed lag regression (Eq. (9)). Because we include contemporaneous effects in computing short-run elasticities, we address endogeneity issues before implementing a particular specification. Moreover, we discuss the potential omitted variable bias that may result from considering only bivariate relationships in estimating elasticities.

By including a contemporaneous effect in the elasticity estimates, we assume that the real wage is exogenous to the vital rates. In the case of the birth rate, this is warranted on the grounds that fertility impacts on labor market conditions with a lag of only about 10 to 15 years, when children acquire substantial working capacity. We can thus safely consider the real wage as being exogenous to the birth rate. Two reasons suggest the same for the death rate. First, variation in mortality was not massive enough to cause relevant fluctuations in labor supply. Specifically, the standard deviation of the rate of natural increase was 5 per 1,000 in 1730–1799 (4 per 1,000 over the whole period), which makes it highly unlikely that mortality impacted on labor supply. Second, nominal wages were highly sticky and adjusted only slowly to shocks in consumer prices (Pfister 2017:711–713, supporting information S2.5). This makes a short-term reaction of wages to demographic events highly unlikely.

Another question relates to whether we should add effects of the respective other vital rate when estimating the elasticity of the birth and the death rates on the real wage. Because fertility depends on parental health and because mortality is also determined by health conditions, fertility possibly reacted on mortality (Lee 1981:363–366). At the same time, infant mortality constituted an important component of the death rate during the period under study, and the number of infant deaths may be influenced by the number of births (Lee 1981:357–358; Weir 1984:37). Consequently, estimates of contemporaneous effects of vital rates may suffer from simultaneity bias due to reverse causation. To resolve this issue, we carry out a Granger causality analysis for the unrestricted VAR (*b*, *d*, *w*) with trend in 1730–1799, using a lag-augmented test suited for multivariate time series with cointegrating relationships (Table 2; cf. Lütkepohl 2007:318–320). The first line in Table 2 corroborates the assumption that the real wage was exogenous to the vital rates. Line 2 suggests that the effect of the birth rate on the death rate via infant mortality is irrelevant in our series. The last two lines, by contrast, imply that the birth rate was endogenous to the death rate and that there may have existed a contemporaneous effect.

**Table 2:** Granger causality tests for the VAR (*b*, *d*, *w*), 1730–1799

	*F*	*df*1	*df*2	*p*
1. *H*_0_: *b* and *d* Do Not Granger-Cause *w*	0.71	4	168	.583
2. *H*_0_: *b* Does Not Granger-Cause (*d, w*)	0.80	4	168	.523
3. *H*_0_: *d* Does Not Granger-Cause (*b, w*)	3.62	4	168	.007
4. *H*_0_: No Instantaneous Causality Between *d* and (*b, w*)	9.03	2		.011

*Note*: VAR has two lags (see the text) and includes a constant and a trend. We apply lag augmented causality test as described by Lütkepohl (2007:318–320) and implemented in JMulti.

*Source*: Own calculation based on series shown in Fig. 3.

In sum, based on theoretical considerations and empirical evidence, we assume that fertility is endogenous to mortality and that the vital rates are endogenous to the real wage. We therefore include a contemporary effect of the real wage into all elasticity estimates, and contemporary as well as lagged effects of the death rate into the estimate of the preventive check. In the case of the positive check, by contrast, bivariate ADL regression is the correct specification.

The final preliminary step concerns the determination of the number of lags. AIC, HQ, SC, and FPE information criteria for bivariate and trivariate VAR almost universally suggest a lag order of 2; hence, all analyses have been carried out with two lags in levels (ADL regressions) and one lag in first differences (ECM).

Table 3 presents the results of an ECM following the Engel-Granger method for the preventive check (cf. Eq. (8)). Coefficients for *w* indicate the elasticity of population with respect to the real wage via an increase in the number of births. To arrive at a more conventional measure that is also comparable with the existing literature, we divide the regression coefficient of the real wage by the arithmetic mean of the birth rate, which yields the point elasticity of the birth rate at the mean. Following our earlier discussion, the error correction equations in the lower panel of Table 3 allow assessing the short-term component of the preventive check. The contemporaneous effect of the change of the real wage (Δ*w*) calculates instantaneous elasticity, and the sum of the contemporaneous and the lagged effects underlies the estimate of cumulative short-term elasticity (see last two lines of Table 3). The cointegrating equation in the upper panel of Table 3, which captures the structural relationship between the birth rate and the real wage, includes both a constant and a trend. The constant follows from Eq. (2), whereas the trend controls for the parallel fall of both the birth rate and the real wage at the beginning of the period. Dividing the coefficient of the lagged real wage (*w* – 1) by the mean of the birth rate gives the long-term elasticity of the birth rate on the real wage.

**Table 3:** Error correction models for the birth rate, 1730–1870: OLS regression coefficients, with standard errors shown in parentheses

	Constant	Trend	*w* – 1	
Cointegrating Equation, Dependent Variable Is *b* – 1^a^				
Coefficient	0.060	–2.6E–05	0.005	
	(1)		(2)	
Error Correction Equations, Dependent Variable Is Δ*b*				
ECT	–0.257*	(0.063)	–0.270*	(0.061)
Δ*b* – 1	–0.232*	(0.077)	–0.237*	(0.077)
Δ*d*			–0.125*	(0.039)
Δ*d* – 1			–0.102*	(0.039)
Δ*w*	0.007*	(0.001)	0.006*	(0.001)
Δ*w* – 1	0.010*	(0.002)	0.008*	(0.002)
*R*^2^, adjusted	.444		.490	
Durbin-Watson statistic	1.90		1.94	
Implied instantaneous elasticity	0.19		0.16	
Implied cumulative short-term elasticity	0.45		0.37	

*Source*: Own calculations based on series shown in Fig. 3.

^a^Implied long-term elasticity of *b* on *w =* 0.13.

*p < .05

We estimate two variants of the coefficients for the short-term relationships (lower panel of Table 3). The first considers only the bivariate relationship between *b* and *w*, whereas the second includes the death rate *d*. This variable controls for the effect of income on fertility via parental health, which is not related to fertility decisions and hence the preventive check per se. The effect of changes in *d* is quite substantial; its inclusion reduces the elasticity estimates by about one-sixth (for England, cf. Bailey and Chambers 1993:357–358). With this correction, the short-term cumulative elasticity of the birth rate on the real wage still amounts to a substantial 0.37. The long-term elasticity implied by the cointegrating relationship in the upper panel of Table 3, by contrast, has a much lower value of 0.13.

**Fig. 4:**
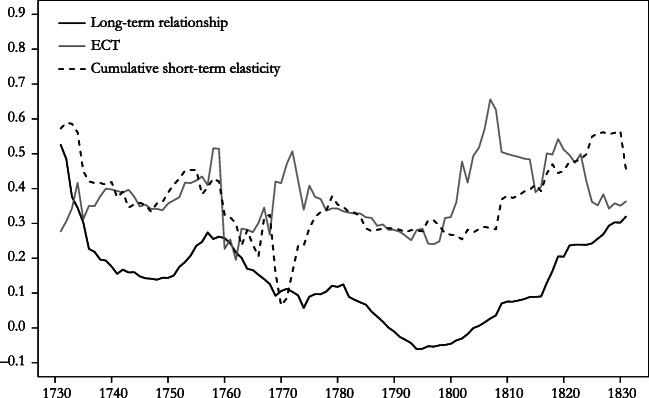
Evolution of the preventive check, 1730–1870: Elasticity of the birth rate on the real wage in rolling 40-year windows (specification 2 of Table 4). The horizontal axis refers to the starting year. *Source*: Own calculation based on the series shown in Fig. 3.

Tests for autocorrelation in residuals (Durbin-Watson, as shown in Table 3; Box and Pierce Portmanteau test over lags 5 to 30) suggest acceptable model adequacy at conventional levels of statistical significance. Because the time series properties of the variables involved change over time—*I*(1) over the whole period and *I*(0) in 1816–1870—parameter estimates may be unstable, however. To explore this possibility, we estimate the second specification of the ECM of Table 3 separately for 40-year rolling windows. Figure 4 displays the trajectory of the main parameters. According to the Johansen trace statistic, the birth rate and the real wage were cointegrated in all time windows until the one beginning in 1818. In 9 of the 13 remaining time windows, the test suggests that the system is full rank: that is, the null hypothesis that rank less than or equal to 1 is rejected at the 10% level of statistical significance. Hence, in Fig. 4, we stop reporting the long-term elasticity and the coefficient of the error correction term from 1819. The disappearance of a long-term relationship between the birth rate and the real wage corroborates the earlier impression of a profound change of demographic mechanisms in the 1810s.

Across the time windows starting before 1819, the long-term elasticity also appears rather unstable; the mean of 0.14 goes together with a standard deviation of 0.12. Figure 4 suggests that the magnitude of the long-term relationship declines notably with the windows starting in the early 1760s (which include values relating to the early 1800s). It thus appears that the shocks of the Napoleonic Wars combined with possibly poor data quality have a negative impact on the stability of the long-run relationship between fertility and the real wage. In the first 30 time windows, all relating to the eighteenth century, the long-run elasticity of *b* on *w* amounted to 0.23, which is not much below the estimate for the cumulative short-term elasticity in Table 3.

The cumulative short-term elasticity of the birth rate on the real wage remained fairly stable over the period under study. The standard deviation across all 101 time windows is 0.11, compared with a mean of 0.36, which is very close to the parameter estimate in Table 3. A similar conclusion holds for the parameter of the error correction term (ECT, or λ in Eq. (8)): the standard deviation of 0.09 across all time windows compares with a mean of 0.38. This is higher than the estimate of 0.26 in Table 4, which suggests this value as a lower-bound estimate of the speed of adjustment of the birth rate to shocks in the real wage.

**Table 4:** Autoregressive distributed lag regressions for the crude death rate: OLS regression coefficients, with standard errors shown in parentheses

	1730–1799		1816–1870
	(1)	(2)	(3)
Constant	0.083^†^ (0.043)	0.015 (0.030)	0.015 (0.012)
Trend	–5.6E–05 (3.4E–05)	2.0E–06 (2.3E–05)	
*d* – 1	0.394* (0.112)	0.469* (0.081)	0.353* (0.146)
*d* – 2	0.097 (0.112)	0.249* (0.076)	0.149 (0.145)
*w*	–0.014* (0.006)	–0.013* (0.004)	0.001 (0.003)
*w* – 1	–0.016^†^ (0.008)	0.001 (0.006)	–0.001 (0.004)
*w* – 2	0.023* (0.006)	0.014* (0.004)	0.000 (0.003)
Year Dummy Variables	No	Yes	No
*R*^2^, Adjusted	.503	.795	.102
Durbin-Watson Statistic	1.97	1.97	2.02
Implied Instantaneous Elasticity	–0.41	–0.38	0.02
Implied Cumulative Elasticity	–0.20	0.06	0.01

*Notes*: Year-specific dummy variables in regression (2) refer to 1740, 1757, 1758, 1764, 1766, 1772, and 1795 (selection based on studentized residuals *r* > 2).

*Source*: Own calculations based on series shown in Fig. 3.

^†^*p* < .10; *p < .05

Let us turn to the positive check. Because the death rate is trend-stationary and the real wage is *I*(1) in 1730–1870, no stable relationship exists between the two variables over the whole period under study (cf. Cuddington and Dagher 2015:197). For this reason, we carry out an exploratory analysis of an autoregressive process in first differences.^3^ A recursive Chow test (but not cumulative sum (CUSUM) test) suggests structural breaks in 1808–1810 and 1814–1817, for which the *F* test statistic exceeds the critical value at the 5% level of statistical significance. This again lends support for the thesis that the beginning of the nineteenth century saw a major change in the nature and the strength of the Malthusian checks.

Because the death rate and the real wage can be considered (trend-)stationary in 1730–1799 and 1816–1870 (see section A of the online appendix), it is possible to estimate ADL regressions in levels for the subperiods before and after the structural break, respectively (Table 4; cf. Eq. (9)). Because there is no long-term structural relationship between the death rate and the real wage, we produce only estimates for the instantaneous and the cumulative short-term elasticities by dividing regression coefficients by the mean of the death rate in the respective period.

During the eighteenth century, quite a strong relationship existed between the real wage and the death rate. According to the baseline specification of column 1 in Table 4, the coefficients of both the contemporaneous and the lagged effects of the real wage are statistically significant, and the instantaneous elasticity of the death rate on the real wage is about –0.4. The cumulative elasticity, which includes the two lagged effects, is much weaker, at –0.2. The difference between the two elasticity measures is due to a positive effect in the second lag, which is actually stronger than the contemporaneous effect in absolute magnitude (0.023 vs. –0.014). A possible explanation is that the survivors of a mortality crisis constitute a population with high resistance to morbidity, which depresses mortality in the years immediately following a crisis.

Spikes in the death rate resulting from food crises and epidemics produce potential outliers that may lead to a violation of the normality assumptions that underlie the coefficient estimates. Whereas Cook’s D does not suggest the presence of influential cases, several studentized residuals of Eq. (1) have values greater than 2, which points to the presence of potential outliers. With the exception of 1764, all cases involve years that were characterized by serious subsistence crises (see note to Table 4 and compare with Fig. 2). When dummy variables are included for these years, the contemporaneous effect of the real wage remains stable, whereas the lagged effects are much reduced (Eq. (2) in Table 4). Hence, the magnitude of the cumulative effect appears strongly influenced by the frequency and severity of subsistence crises.

**Fig. 5:**
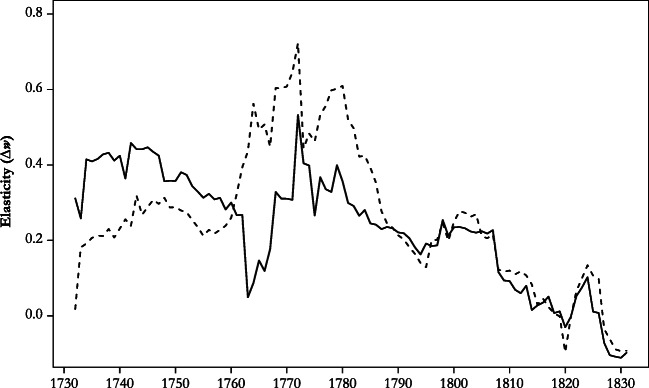
Evolution of the positive check, 1730–1870: Elasticity of crude death rate on the real wage in rolling 40-year windows (from Eq. (1) of Table 3). The solid line represents implied instantaneous elasticity; the broken line represents implied cumulative elasticity. Signs are inverted. The horizontal axis refers to the starting year. *Source*: Own calculation based on the series shown in Fig. 3.

The last column of Table 4, which relates to the subperiod 1816–1870, suggests that the positive check had ceased to exist after 1815. None of the coefficients of the real wage are statistically significant, and both elasticity measures are close to 0. We would like to know, of course, whether the positive check disappeared suddenly at the beginning of the nineteenth century—as the chronology of excess mortality in Fig. 2 and the results of the Chow test for structural breaks suggest—or more gradually over a longer period. Therefore, in analogy to our procedure in case of the preventive check, we estimate Eq. (1) of Table 4 for shorter periods of 40 years and roll these time windows across the whole period of observation (Fig. 5). Very tentatively, this exercise suggests that the instantaneous elasticity of the death rate on the real wage declined slowly after the 1740s. Windows including both crises of the early 1770s and the second half of the 1800s saw a temporary reversal of this trend, however. With the time windows starting around 1810, the elasticity of *d* on *w* fell to 0, which is consistent with the result of the Chow test for structural breaks. We conclude that the positive check disappeared around 1810, but it is possible that its strength declined gradually already in the course of the second half of the eighteenth century.

## Discussion

The results of the two previous sections can be summarized under three headings: (1) we find a roughly stable preventive check; (2) there is evidence of an unstable Malthusian situation in the eighteenth century; and (3) the 1810s saw a transition to a post-Malthusian regime. What follows further characterizes these results, places them in a wider research context, and explores possible explanations for the emergence of an unstable Malthusian situation in the eighteenth century and the transition to the post-Malthusian era in the early nineteenth century.

### The Preventive Check

From a methodological perspective, we find, first, both contemporaneous and lagged effects of the real wage on the crude birth rate (cf. Dribe and Scalone 2010). Second, we show that it is important to control for the contemporaneous and lagged effects of mortality on fertility, which captures unobserved effects of income variation on fertility via parental health. This channel involves an element of the positive check, and it should therefore be removed when assessing the magnitude of the preventive check. Third, using an error correction mechanism framework with contemporaneous effects, our investigation is the first to estimate the strength of the preventive check both in the long term and the short term; recent studies on other countries focused on either long-term (Klemp and Møller 2016:858; Møller and Sharp 2014:129) or short-term elasticities (e.g., Edvinsson 2017; Fernihough 2013).

As for substance, we find that the preventive check measured by the short- and long-term elasticity of the crude birth rate on the real wage was of an order of magnitude of 0.2 to 0.35. The lower value refers to the long-run elasticity during the eighteenth century; the higher value, to the cumulative short-run elasticity. There is no evidence that the strength of the short-term association changed over time. During the eighteenth century, changes in fertility absorbed about 25% of an income shock per year, as indicated by the error correction term. A similar speed of adjustment via the preventive check was obtained for England (Møller and Sharp 2014:126, second and fourth lines of α matrix). Our results for the magnitude of the preventive check are also comparable with values found for England (Møller and Sharp 2014:218–219), northern and central Italy (Fernihough 2013:329), and Sweden in 1630–1720 (Edvinsson 2017:217). During the period 1721–1870, which roughly corresponds to the period analyzed in this study, the short-term preventive check was stronger in Sweden, however (0.52 to 0.63). With the exception of the latter result, it thus appears that an elasticity of the crude birth rate on the real wage at the order of magnitude of 0.2 to 0.35 corresponds to a widespread and fairly stable pattern in the centuries prior to the onset of the first fertility transition. Although not particularly strong, the preventive check was thus present in pre-modern Europe, and it may have operated not only through the marriage rate but also through intramarital fertility control (Cinnirella et al. 2017; Dribe and Scalone 2010).

### Unstable Malthusian Situation in the Eighteenth Century

In Germany, vital rates and the real wage did not constitute a coherent system during the period under study. Whereas the real wage and the birth rate both had a unit root and were cointegrated, the death rate was stationary or trend-stationary. Consequently, there was no long-term relationship between income and mortality. To be sure, eighteenth century Germany (1730–1799) corresponded to a Malthusian situation in that demographic expansion went together with a strong decline of the real wage; population and material welfare were closely linked. Moreover, the death rate reacted strongly to income shocks in the short run: the instantaneous elasticity of the death rate on the real wage (based on the contemporaneous effect) was high, at –0.4. Only research for Sweden shows a higher value for the positive check in the short run; in England and in Italy, the elasticity of the crude death rate on the real wage was less than –0.3 (Edvinsson 2017:217; Fernihough 2013:329; Møller and Sharp 2014:218–219). The combination of a strong short-term positive check with the absence of a long-term relationship between the death rate and real wage is consistent with a pessimistic view whereby “famine seems to be the last, the most dreadful resource of nature” to remedy for the “vices of mankind” (Malthus 1798/1998:44). In sum, eighteenth century Germany was characterized by Malthusian disequilibrium in that the real wage could fall considerably without effective adjustment taking place, which went to together with a high vulnerability to short-term supply shocks.

The absence of the positive check in the long run implies that the level of mortality was driven by exogenous forces. The contrast with respect to the birth rate is plausible in the sense that decisions of couples have some influence on marriages and births, whereas deaths depend on factors exogenous to household decisions, such as the epidemic environment and public health provision.

Specifically, the exogenous nature of eighteenth century mortality is signaled by the fact that whereas the real wage declined at an annual rate of –0.5% in 1730–1799, the death rate also fell at 2 per 1,000 per annum. From a Malthusian perspective, we would expect that a decline of income adversely affects health conditions, which should raise the death rate in the long run. Malthusian disequilibrium in the sense of a movement of population and the real wage in opposite directions over more than half a century was exacerbated by the incapacity of the preventive check to compensate for the absence of the positive check in the long run. The figures mentioned previously imply that the birth rate compensated merely for about one-quarter of the fall of the real wage; although it declined slightly over the period 1730–1799 (–0.12% per annum), the reduction fell far short of effectively counteracting the negative effect of population growth on the real wage.

Given the current state of knowledge, three candidate explanations exist for the development of a Malthusian disequilibrium in circa 1730–1799. The first relates to a change in the epidemic environment, which led to a reduction in the level and the volatility of mortality between the 1640s and the early nineteenth century. Beginning with the 1640s, the virulence of bubonic plague and related epidemics receded, and these disappeared altogether in the early eighteenth century, which explains the existence of a positive rate of natural increase already at the beginning of our period under study (Eckert 1996:6, 159–160; Wahrmann 2012). Waves of other epidemic diseases also seem to have lost their force after the Seven Years’ War (1756–1763), and the widespread adoption of smallpox vaccination shortly after 1800 brought a final reduction in the mortality rate in the pre-modern age (Gehrmann 2000:137–138, 164–165, 286–291).

Second, German grain markets became mor0e integrated between circa 1650 and the late eighteenth century (Albers et al. 2018). This, together with global warming after the end of the Maunder minimum (circa 1640–1710), reduced the risk that food stocks ran out in years of poor harvests, which dampened the negative effect of an income shock on food consumption. In combination, climate change and market integration provide a potential explanation for the tendency of the short-term positive check to weaken during the second half of the eighteenth century.

Third, the expansion of the nonagricultural sectors, particularly in the form of regional export industries or proto-industries, mitigated the effect of declining marginal output in agriculture on household income. The relative dynamic of nonagricultural sectors is testified by the rise of the share of employment outside agriculture and by the fact that foreign trade expanded faster than population despite stagnant income per working day (Pfister 2011:5, Pfister 2015). Before the rise of centralized factory production, which had to await the second half of the nineteenth century in many branches of German industry, households frequently engaged in both agriculture and manufacture production (e.g., Quataert 1985; for a general discussion, see de Vries 2008). Whereas harvest failures affected lower-class households in purely agricultural regions also through a fall in exchange entitlements (Sen 1981) as a consequence of a decline of employment in harvest work and threshing, revenues from nonagricultural activities stabilized household incomes during food crises in regions with sizable nonagricultural sectors (Bass 1991:27, chapters 5 and 6), which contributed to the decline of volatility in mortality and, possibly, of the short-term positive check. Moreover, by drawing on resources of idle labor during seasons with low activity in agriculture, work in regional export industries stabilized household income and thereby mitigated the negative health effects of declining earnings per day. Taken together, the second and third explanations suggest that market creation supported the maintenance and widening of a Malthusian disequilibrium in the eighteenth century.

### Transition to the Post-Malthusian Era in the Early Nineteenth Century

In the late 1800s and 1810s, Germany’s demographic regime changed quite suddenly: the short-term positive check disappeared, and harvest failures were not associated with a negative rate of natural increase on the national level after 1814. The long-term relationship between the birth rate and the real wage also ceased to exist. Only the short-term preventive check remained in effect beyond the 1810s. Finally, the rate of population growth doubled from 0.4% to 0.8% per annum.

From a systemic perspective, both vital rates and the real wage were stationary in the period 1816–1870, whereas population was trend-stationary, suggesting a state of post-Malthusian equilibrium. According to this model, real wage equilibrium was not brought about by the interaction of the positive and preventive checks—that is, by Malthusian adjustment—but rather by a constant rate of (unmeasured) technological progress, *g*^∗^, that equaled the constant rate of natural increase of population times the elasticity of the real wage on population, *g*^∗^ = β(*c*_1_ − *c*_2_) (cf. Issues in the Analysis of Malthusian Systems section). Thus, on the aggregate level, there apparently existed a linear relationship between the level of technology and population size; technological progress depended on changes in scale. Note that there is no theoretical reason why the equality *g*^∗^ = β(*c*_1_ − *c*_2_) should hold; at this point, the equilibrium we observe appears fortuitous.

The massive acceleration of population growth at the beginning of the nineteenth century is consistent with the notion that in the early stages of economic growth, technology was labor-biased rather than skill-biased, so that technological progress had mainly the effect of loosening parents’ budget restrictions on raising children. However, the finding that technology was related to population size is slightly at variance with the unified growth literature, which presumes that the rate of technological *change* (rather than the level of technology) depends on population size (Galor 2011; Galor and Weil 2000; Kremer 1993).

Scale-dependent technology can be conceived either as Boserupian growth (Boserup 1965) or as Smithian growth (Kelly 1997). Several processes taking place in early nineteenth century Germany are consistent with both types of scale-related growth and provide potential explanations for the disappearance of the positive check.

Boserupian growth is suggested by the spread of innovations linked with the first phase of agricultural modernization. These new techniques included stall-feeding and the cultivation of fodder crops and the potato, all of which were labor-intensive. The spread of potato cultivation, which gained in momentum around 1800, was of particular importance in at least two respects. On the one hand, it went together with an increase in food output per acre and thus created room for an acceleration of population growth (Nunn and Qian 2011). On the other hand, potatoes react to seasonal weather conditions in different ways than grain, rendering a mix of grain and potato farming a better way to diversify food risk than grain farming alone (Uebele and Grünebaum 2013). Consequently, the spread of potato cultivation constitutes a potential explanation for the disappearance of the positive check in the early nineteenth century. Because potato cultivation was highly labor-intensive, the Malthusian disequilibrium of the eighteenth century, which led to a vast expansion of the labor supply, may have been pivotal for its widespread adoption, as posited by Boserup (1965).

Smithian growth results from a deepening of labor division. An increase in the spatial division of labor promotes regional specialization according to comparative advantage, which raises the technological efficiency of a regional economy. Enhanced specialization of individual workers increases their task-specific skills, and this promotes learning and the production of technological innovation. In the Smithian perspective, the extent of labor division is driven by the size of the market, which in turn is determined by population size and by trade costs. Already during the eighteenth century, several processes mentioned earlier—grain market integration, development of regional export industries, and the expansion of external trade—represent aspects of Smithian growth. During the era between the Napoleonic Wars and national unification, Smithian growth gained in momentum not only because of rapid population growth but also as a result of changes in institutions and transport technology that lowered trade costs. Institutional change that promoted market integration included the creation of modern states with relatively large internal markets at the beginning of the century and the foundation of a customs union in 1834 (Albers and Pfister 2018; Keller and Shiue 2014). Reductions in transport costs followed from programs of public roads construction in the first part of the nineteenth century and from the expansion of railway networks from the 1840s (Hornung 2015; Uebele and Gallardo Albarrán 2015). On the one hand, grain market integration resulting from these processes reduced the risk of local food shortages, which contributes to explaining the disappearance of the positive check in the 1810s. On the other hand, the processes mentioned rendered it possible to realize the potential of rapid population growth for market extension and hence for increasing the level of technology more or less continuously.

## Conclusion

Combining novel data sets on vital rates and the real wage, we establish Germany’s trajectory out of a premodern Malthusian regime in the one and a half centuries prior to national unification in 1871. Specifically, we find that in 1816–1870, Germany was in a post-Malthusian regime where income still had a positive impact on population growth through the short-term preventive check, but where a positive relationship between the level of technology and population fully compensated for the negative impact of population size on material welfare. The transition to the post-Malthusian era was predated by Malthusian disequilibrium in the eighteenth century: whereas the death rate reacted strongly to income shocks in the short term, there was no equilibrium relationship between the real wage and the death rate in long run. Because the birth rate adjusted only partially for income fluctuations, population could thus expand despite a fall of the real wage. The resulting population growth may have paved the ground for a post-Malthusian pattern of development where the rate of increase of the level of technology depended on population size, as suggested notably by a Boserupian perspective (Boserup 1965). The finding that the transition to the post-Malthusian era took place quite suddenly in the late 1800s and 1810s also suggests a role for the political shocks resulting from the Revolutionary and Napoleonic Wars around 1800. These created a fertile ground for institutional change and infrastructure development that promoted Smithian growth.

Our results highlight the variability of country-specific experiences regarding the demographic aspects of the first phase of modern development. Important formulations of unified growth theory hypothesize a positive relationship between population size and the rate of technological change. Over time, the parallel increase in population size and the rate of technological change lead to a situation in which the Malthusian checks do not fully adjust to the rise in real income anymore. Incomplete adjustment engenders a transition to a post-Malthusian era where population can grow rapidly without a decline in material welfare. The English experience from the seventeenth century may correspond to such a smooth transition. In Germany, the post-Malthusian era from the 1810s to the 1870s was characterized by strong population growth and roughly constant real wages, which suggests a positive correlation between population and the level of technology. The existence of a Malthusian disequilibrium in the eighteenth century, characterized by a combination of a falling real wage, rising population, and a positive check operating solely through short-term shocks casts doubt on the role of a rising rate of technological change as the main characteristic of the late Malthusian era preparing the transition to the post-Malthusian era. Rather, shocks that took place at the beginning of the nineteenth century and whose nature remains to be explored in greater detail were pivotal in making scale effects of population on technology effective.

## Electronic supplementary material

ESM 1 (PDF 85 kb)

## Footnotes

1 Already in 1800–1815, the trend growth rate was 0.8% per annum, but see the earlier caveats concerning data quality during the war period.

2 For the subperiod 1730–1799, the trace test statistic for the pair (*d*, *w*) is slightly below the critical value for the 5% level of statistical significance. For the pair *(b*, *d*) in 1730–1799, the information criteria yield ambiguous results (Akaike information criterion (AIC) and final prediction error (FPE) 4; HQ Hannan and Quinn criterion (HQ) and SC Schwarz criterion(SC) 2). With a lag order of 4, the trace statistic for *r* ≤ 1 is 17.96, which exceeds the critical value at the 1% level.

3 This corresponds to Eq. (8) without the error correction term. A dummy variable was included to control for the potential outlier in 1772.
