# Supplementary material for: From Malthusian Disequilibrium to the Post-Malthusian Era: The Evolution of the Preventive and Positive Checks in Germany, 1730–1870
Source: Demography. 2020 May 4;57(3):1145–70. doi: 10.1007/s13524-020-00872-w (PMC7329779; doi:10.1007/s13524-020-00872-w)
Supplement: Supplementary file 1 — (PDF 85 kb) [file 13524_2020_872_MOESM1_ESM.pdf]

Online Appendix

Evolution of the Preventive and Positive Checks in Germany, 1730–1870
Ulrich Pfister and Georg Fertig

A. Unit Root and Stationarity Tests

We run ADF and KPSS tests on the vital rates and the log of the real wage (Table A1). A statistically significant ($p<.05$) value of an ADF test rejects the null hypothesis that there is a unit root in the series. A significant KPSS test rejects the null hypothesis that the series is stationary. For all series, we start testing the untransformed (level) series, and proceed to models with trend and then to first-differenced series. This is done for ADF tests until the unit root hypothesis is rejected, and for KPSS tests until they fail to reject the stationarity hypothesis.

Lag order for ADF tests was determined using AIC, HQ, SC and FPE selection criteria. When results were ambiguous, AIC and SC criteria suggested the highest and the lowest lag orders. In these cases, we carried out tests with these alternative lag orders. The results are as follows:

The crude birth rate is either stationary (ADF) or is integrated at $I(1)$ (KPSS) both in the whole period under study and in 1730–99. In the analysis above we assume $I(1)$. For 1816–70, KPSS suggests $I(0)$, whereas ADF is ambiguous. We assume that the birth rate was stationary during the second sub-period.

The crude death rate is either stationary (ADF) or trend-stationary (KPSS) both in the whole period under study and in 1730–99. Since the series has a statistically significant negative trend in both periods (-2 per thousand p. a.) we assume trend-stationarity. Similarly, in 1816–70 the series is stationary according to the ADF test, whereas the KPSS test suggests either trend-stationarity or $I(1)$. Further analysis shows that changes in infant mortality impacted on the behavior of the death rate in 1816–70. The infant mortality rate rose during the third quarter of the nineteenth century, and this series is clearly $I(1)$ in 1826–1870 (Gehrmann 2011: 849, series b). If we eliminate the non-infant death rate from the crude death rate following Weir (1984: 37, note 26, but using annual data for infant mortality from 1826) then this modified series is clearly stationary in 1816–70. We thus assume the death rate to be stationary in the second sub-period but are aware that we thereby abstract from the structural worsening of the living conditions of infants from about 1850.

For the real wage the majority of tests suggest $I(1)$ for the whole period. In the first sub-period, the series is trend-stationary, in 1816–70 it is probably stationary (the KPSS test is inconclusive).

Reference

Gehrmann, R. (2011). Infant mortality in Germany in the nineteenth century. *Comparative Population Studies—Zeitschrift für Bevölkerungswissenschaft, 36,* 839–868.

Table A1 Unit root and stationarity tests

	ADF			KPSS		
	Lag	Test statistic	p	Truncation lag	Test statistic	p
1730–1870						
Crude birth rate						
level	3 (AIC)	-4.61	<0.01	2	1.288	<0.01
level+trend				2	0.161	0.038
1st differences				2	0.069	>0.1
Crude death rate						
level	1 (AIC)	-4.47	<0.01	2	2.778	<0.01
level+trend				2	0.093	>0.1
Real wage (logs)						
level	6 (AIC)	-2.40	0.166	2	0.636	0.019
level	3 (SC)	-2.87	0.052	8	0.280	>0.1
level+trend	6 (AIC)	-2.32	0.445	2	0.607	<0.01
level+trend	3 (SC)	-2.78	0.253	8	0.267	<0.01
1st differences	5 (AIC)	-6.44	<0.01	2	0.064	>0.1
1st differences	3 (SC)	-7.25	<0.01	8	0.155	>0.1
1730–1799						
Crude birth rate						
level	2 (AIC)	-4.05	<0.01	1	0.740	<0.01
level+trend				1	0.353	<0.01
1st differences				1	0.085	>0.1
Crude death rate						
level	1 (AIC)	-4.66	<0.01	1	0.645	0.018
level+trend				1	0.067	>0.1
Real wage (logs)						
level	2	-2.29	0.211	1	2.162	<0.01
level+trend	2	-3.91	0.019	1	0.101	>0.1
1816–1870						
Crude birth rate						
level	2	-2.88	0.057	1	0.229	>.1
level+trend	2	-2.94	0.202			
1st differences	2	-6.37	<.01			
Crude death rate						
level	1	-3.28	0.022	1	0.528	0.035
level+trend				1	0.143	0.054
1st differences				1	0.027	>.1
Real wage (logs)						
level	2	-4.05	<.01	1	0.166	>.1

Source: Own calculation based on series shown in Figure 3.

B. Population and vital rates

Table B1 Population (1690–1870) and vital rates (1730–1870)

Year	Population (1000)	Crude birth rate	Crude death rate
1690	10634.1		
1691	10628.1		
1692	10573.6		
1693	10564.5		
1694	10559.6		
1695	10705.3		
1696	10934.8		
1697	11170.7		
1698	11353.7		
1699	11482.5		
1700	11718.7		
1701	11881.9		
1702	11864.9		
1703	11952.0		
1704	12028.7		
1705	12093.9		
1706	12183.9		
1707	12341.5		
1708	12435.4		
1709	12528.5		
1710	12548.4		
1711	12667.8		
1712	12724.0		
1713	12814.6		
1714	12911.6		
1715	13021.8		
1716	13262.5		
1717	13395.7		
1718	13259.1		
1719	13219.2		
1720	13163.3		
1721	13107.6		
1722	13258.8		
1723	13503.9		
1724	13533.6		
1725	13741.9		
1726	13906.9		
1727	13835.2		
1728	13689.2		

1729	13583.1	0.043	0.054
1730	13696.4	0.043	0.039
1731	13736.4	0.044	0.042
1732	13885.0	0.047	0.037
1733	13980.2	0.044	0.039
1734	14165.1	0.046	0.034
1735	14257.4	0.039	0.034
1736	14382.4	0.040	0.033
1737	14440.2	0.038	0.035
1738	14541.9	0.040	0.034
1739	14651.9	0.040	0.034
1740	14641.5	0.038	0.040
1741	14662.0	0.035	0.036
1742	14764.9	0.038	0.033
1743	14895.6	0.040	0.033
1744	15058.2	0.038	0.030
1745	15202.7	0.037	0.030
1746	15324.7	0.039	0.033
1747	15422.5	0.036	0.031
1748	15496.6	0.036	0.034
1749	15567.9	0.036	0.034
1750	15588.6	0.034	0.035
1751	15639.1	0.036	0.032
1752	15735.2	0.036	0.029
1753	15844.0	0.037	0.029
1754	15955.8	0.037	0.029
1755	16065.8	0.036	0.029
1756	16168.3	0.036	0.029
1757	16065.2	0.034	0.040
1758	15916.4	0.031	0.041
1759	15858.1	0.035	0.038
1760	15861.1	0.038	0.038
1761	15819.0	0.036	0.038
1762	15750.8	0.036	0.040
1763	15660.6	0.035	0.040
1764	15813.3	0.040	0.030
1765	15961.4	0.039	0.030
1766	16069.5	0.040	0.032
1767	16180.3	0.040	0.032
1768	16297.5	0.037	0.029
1769	16460.6	0.040	0.029
1770	16595.8	0.038	0.029
1771	16632.0	0.035	0.033
1772	16368.2	0.031	0.048
1773	16372.4	0.035	0.035

1774	16523.2	0.039	0.030
1775	16659.5	0.038	0.030
1776	16779.3	0.037	0.030
1777	16922.8	0.038	0.030
1778	17054.2	0.039	0.031
1779	17174.3	0.037	0.030
1780	17387.1	0.040	0.028
1781	17486.5	0.038	0.032
1782	17589.8	0.037	0.031
1783	17668.0	0.037	0.033
1784	17751.8	0.036	0.032
1785	17864.0	0.037	0.030
1786	17940.3	0.037	0.033
1787	18053.1	0.036	0.030
1788	18173.9	0.037	0.030
1789	18268.5	0.036	0.031
1790	18369.4	0.036	0.030
1791	18524.8	0.038	0.029
1792	18690.6	0.039	0.029
1793	18782.0	0.035	0.030
1794	18836.2	0.036	0.033
1795	18790.1	0.035	0.037
1796	18859.3	0.037	0.033
1797	18959.7	0.039	0.033
1798	19139.6	0.038	0.028
1799	19277.8	0.040	0.032
1800	19362.4	0.038	0.033
1801	19497.8	0.039	0.030
1802	19725.3	0.041	0.028
1803	19842.3	0.038	0.030
1804	20046.5	0.038	0.027
1805	20149.9	0.037	0.030
1806	20310.9	0.037	0.031
1807	20367.2	0.038	0.038
1808	20410.4	0.036	0.036
1809	20507.9	0.033	0.031
1810	20717.6	0.036	0.028
1811	20868.5	0.037	0.032
1812	21107.7	0.036	0.027
1813	21227.3	0.034	0.031
1814	21265.8	0.034	0.035
1815	21596.9	0.038	0.025
1816	21838.0	0.036	0.026
1817	22028.3	0.034	0.027
1818	22221.0	0.033	0.027

1819	22433.1	0.037	0.028
1820	22699.9	0.036	0.026
1821	23017.0	0.037	0.024
1822	23316.1	0.036	0.025
1823	23608.8	0.036	0.025
1824	23906.8	0.036	0.025
1825	24218.4	0.036	0.025
1826	24498.1	0.036	0.027
1827	24744.0	0.034	0.026
1828	24954.2	0.034	0.026
1829	25168.8	0.034	0.027
1830	25389.5	0.034	0.027
1831	25566.4	0.033	0.028
1832	25702.5	0.033	0.029
1833	25902.4	0.036	0.028
1834	26108.8	0.037	0.031
1835	26361.0	0.036	0.027
1836	26609.4	0.036	0.027
1837	26781.3	0.035	0.030
1838	26987.9	0.036	0.027
1839	27185.9	0.036	0.028
1840	27399.3	0.036	0.027
1841	27653.4	0.036	0.027
1842	27913.1	0.037	0.028
1843	28134.5	0.035	0.028
1844	28400.8	0.035	0.026
1845	28711.1	0.037	0.027
1846	28945.1	0.036	0.028
1847	29023.8	0.033	0.029
1848	29109.7	0.033	0.029
1849	29372.7	0.038	0.028
1850	29605.3	0.037	0.027
1851	29838.7	0.036	0.026
1852	29986.4	0.035	0.028
1853	30069.2	0.034	0.027
1854	30142.4	0.033	0.027
1855	30140.0	0.031	0.028
1856	30358.6	0.033	0.026
1857	30582.5	0.035	0.028
1858	30844.7	0.036	0.028
1859	31146.6	0.037	0.027
1860	31563.4	0.037	0.023
1861	31811.0	0.035	0.027
1862	32133.0	0.035	0.026
1863	32492.9	0.037	0.027

1864	32854.0	0.038	0.028
1865	33067.8	0.037	0.029
1866	33213.8	0.037	0.032
1867	33463.4	0.036	0.027
1868	33692.5	0.037	0.028
1869	33985.5	0.038	0.027
1870	34283.6	0.038	0.028
